# Childhood Environment and Mental Wellbeing at Age 60-64 Years: Prospective Evidence from the MRC National Survey of Health and Development

**DOI:** 10.1371/journal.pone.0126683

**Published:** 2015-06-01

**Authors:** Mai Stafford, Catharine R. Gale, Gita Mishra, Marcus Richards, Stephanie Black, Diana L. Kuh

**Affiliations:** 1 MRC Unit for Lifelong Health and Ageing at UCL, London, United Kingdom; 2 MRC Lifecourse Epidemiology Unit, University of Southampton, Southampton, United Kingdom; 3 Centre for Cognitive Ageing and Cognitive Epidemiology, Department of Psychology, University of Edinburgh, Edinburgh, United Kingdom; 4 School of Population Health, University of Queensland, Brisbane, Australia; University of Granada, SPAIN

## Abstract

**Background:**

Mental wellbeing, conceptualised as positive affect, life satisfaction and realisation of needs that contribute to psychological growth, captures more than the absence of mental ill health. Several nations now aim to monitor and improve mental wellbeing. Whilst many studies document associations between adverse childhood experiences and mental disorders in adulthood, possible links between childhood experiences and adult mental wellbeing have so far received less attention.

**Methods:**

Using data from 1976 men and women in the MRC National Survey for Health and Development, we investigated prospective associations between childhood socioeconomic and psychosocial environments and the Warwick Edinburgh Mental Wellbeing Scale, designed to capture both hedonic and eudaimonic facets of wellbeing, at age 60-64.

**Results:**

Whilst there was no evidence that childhood socioeconomic circumstances were related to later wellbeing independently of other childhood experiences, elements of childrearing and parenting, parental health and adjustment, and childhood illness were related. More advantaged socioeconomic position was associated with greater wellbeing but this did not explain the links between these childhood exposures and adult wellbeing, suggesting alternative explanatory pathways should be considered.

**Conclusions:**

Childhood illness and family psychosocial environment are associated with mental wellbeing in early older age, with effects sizes that are larger or comparable to socioeconomic circumstances in adulthood. Initiatives to improve the nation’s mental wellbeing that include programmes targeted to supporting families and children may additionally have benefits that continue into older age.

## Background

Mental wellbeing is multidimensional and opinion remains divided as to which dimensions should be part of this construct [1]. Its varying definitions have included the hedonic emphasis on wellbeing operationalised through measures of positive affect and happiness, and the eudaimonic emphasis on wellbeing as the realisation of needs that contribute to human psychological growth [2]. Recent evidence links mental wellbeing to a range of indicators of healthy ageing highlighting its importance in later life [3–6]. A life course approach to understanding mental wellbeing would suggest that circumstances and experiences across life may affect wellbeing in older age.

Life course studies have highlighted the relevance of adverse childhood experiences for psychological morbidity in adulthood and older age [7–12] but associations between childhood experiences and adult mental wellbeing have so far received much less attention. Narrative reviews provide evidence for differences in life satisfaction, happiness and positive psychological functioning in adulthood by demographic and socioeconomic factors, health status, social networks and relationship satisfaction, occurrence of life events, psychosocial resources, and personality [2,13–16] though these are primarily focused on exposures captured in adulthood either concurrently with or proximally to mental wellbeing. Among the studies that have considered childhood experiences, the majority have investigated childhood socioeconomic circumstances. Three showed that greater disadvantage (based on parental education, parental occupation or household crowding) was associated with poorer mental wellbeing in adulthood [17–19] but one other did not find such an association [20]. Others have focused on parenting style and showed that parenting characterised by responsiveness and warmth and absence of over-protection was associated with better mental wellbeing [21–23]. Deindl and colleagues [19] recently examined a somewhat wider range of childhood circumstances using data from the Survey of Health, Ageing and Retirement in Europe. In addition to crowding, mentioned above, associations were found between life satisfaction and family composition, cultural capital and health. However, the study relied on retrospective self-reports. To our knowledge, only one prospective study has considered the potential impact on wellbeing of influences measured in childhood across multiple domains [24]. That study used the British Cohort Study to show that family economic circumstances and child’s emotional health and, less importantly, cognitive performance independently predicted life satisfaction at age 34. Family psychosocial factors (measured by maternal emotional health, parental divorce and birth within marriage) did not predict life satisfaction independently but were mediated through the child’s emotional and cognitive health. In the current study we explore the family environment in more detail and consider the importance of the family economic and psychosocial environment for mental wellbeing in early older age.

Based on the literature relating childhood experiences to adult psychological morbidity, it is clear that studies of the long-term consequences of the childhood environment for mental wellbeing need to consider simultaneously a range of indicators capturing characteristics and experiences of the parents, family and child. These include poverty and material disadvantage [25], parental loss [26–28], parental care and supervision [25–29], parental mental health and substance use [27,28–31], and family conflict [7,25]. Clearly, these dimensions of disadvantage are inter-related [32,33]. Studies of multiple childhood exposures in birth cohorts from New Zealand [25] and the United Kingdom [28] capture six general domains: family social disadvantage; family economic disadvantage; impaired child-rearing and parenting; family instability; problems of parental adjustment; and problems in the antenatal and perinatal period. The specific indicators capturing these domains vary from study to study but we consider this to be a useful framework to organise our analysis. Children exposed to these disadvantages have poorer emotional control and lower interpersonal competence [34]. If these emotional and social functioning deficits persist into adult life, it is possible that these will translate into poorer mental wellbeing in old age.

A recently developed instrument, the Warwick Edinburgh Mental Wellbeing Scale (WEMWBS), comprised of positively worded items to capture aspects of positive mental health in population samples, was included in the latest data collection of Britain’s oldest birth cohort study, the MRC National Survey of Health and Development (NSHD), thus allowing prospective examination of the long-term implications of early childhood circumstances into older age. Identification of circumstances and experiences that are predictive of later mental wellbeing will contribute to the ongoing development of interventions to promote healthy ageing that start early on in life. This study therefore aims to examine multiple domains of childhood adversity and their association with positive mental wellbeing in a nationally representative sample of people aged 60–64 years. It also assesses whether adult socioeconomic circumstances mediate these associations.

## Methods

### Study population

The NSHD [35] was originally established to investigate the cost of childbirth and the quality of associated care in the immediate post-war years. A sample of 5,362, comprising all single births within marriage to families of non-manual and agricultural occupation and a random one in four sample of single births within marriage to families of manual occupation, was recruited from all births registered in one week of March 1946 in England, Wales and Scotland. Data have been collected on 23 occasions from birth with the latest data collection in 2006–11 at which time comparison with data from the 2001 England census suggested the cohort was broadly comparable on several socioeconomic characteristics [36]. Ethical approval was obtained from the Greater Manchester Local Research Ethics Committee and the Scotland A Research Ethics Committee. All study members provided informed, written consent to participate in 2006–11.

### Wellbeing at age 60–64

Study members self-completed the 14-item WEMWBS in a postal questionnaire administered at age 60–64. The scale captures positive affect, satisfying interpersonal relationships and positive functioning. Items are worded positively and respondents are asked to indicate how frequently, on a five point scale, they have experienced each statement over the last two weeks. Statements include “I’ve been feeling good about myself”, “I’ve been feeling close to other people”, “I’ve been interested in new things” and “I’ve been feeling optimistic about the future”. Validation work indicates good construct validity for a single factor structure as well as good criterion validity and test-retest reliability and supports its use in general population samples [37]. Where 3 or fewer items were missing, the individual’s mean for complete items was imputed. Items loaded on a single component and were summed using equal weights to create scores which theoretically ranged from 14 to 70 with 70 indicating highest wellbeing.

### Exposure measurement

The following indicators were used to capture the childhood and adolescent environment across multiple domains. The selection of indicators was based on previous work [25,28] but, since our outcome of interest is positive wellbeing, we sought to capture aspects of the environment on a continuum where appropriate rather than maintain a focus on dichotomising adverse exposures. We elected to combine social and economic circumstances. *Family socioeconomic circumstances* were captured by father’s occupational social class at age 4 (or ages 7 or 11 if missing); parental educational attainment; lack of household amenities at ages 2 and 11; crowding at birth and ages 2, 4, 6, 8, 11; mother or father aged under 20 at birth of study member. Children born outside marriage were not included in the original sampling frame for NSHD. *Childrearing and parenting* were captured by whether the study member was breastfed; health visitor-rated mother’s care of the house and child at age 4; teacher-rated parental interest in the child’s education through primary and secondary school; four scales from the Parental Bonding Instrument administered retrospectively at age 43 (maternal and paternal care, maternal and paternal overprotection).

In this cohort, previous factor analysis of the Parental Bonding Instrument items demonstrated two dimensions of parenting which have been referred to as parental care (13 items, alpha = 0.93 for father and 0.91 for mother) and parental overprotection (11 items, internal consistency Cronbach’s alpha = 0.83 for father and 0.83 for mother) [28]. *Family instability* was captured by parental divorce by age 16, separation from the mother before age 6 for 4 or more weeks; number of residential moves by age 16, number of changes of school by age 16. We broadened the parental adjustment domain to additionally include parental health. *Parental health and adjustment* were captured at age 15 by maternal reports of the health of the mother and father; mother’s neuroticism [38]. We broadened the domain of antenatal and postnatal problems to additionally consider illnesses throughout childhood as well as indicators of mental wellbeing in childhood. *Child health and wellbeing* was captured by birth weight; total weeks absent from school due to illness ages 6–12; teacher-rated happiness and sociability at ages 13 and 15.

### Covariates

We aimed to identify exposures in childhood that may be related to wellbeing at age 60–64. Testing of all possible mediating pathways was beyond the scope of the current study. However, we did test whether associations remained on adjustment for social class in adulthood, since previous work suggests there may be both latent and pathway effects of childhood social class [39]. Head of household occupational social class at age 53 (or age 43 if missing (n = 95)) was used as an indicator of highest attained socioeconomic position. Concurrent social class was not used because a substantial number of study members had retired from their main job by age 60–64.

### Statistical analysis

WEMWBS scores were normally distributed (mean 52, median 52, standard deviation 8) and linear regression was used to estimate the mean difference in WEMWBS score for each childhood exposure considered one at a time (in bivariate analysis: Models M1). Next, multiply adjusted linear regression models assessed associations between each exposure adjusted for the other childhood exposures within the same domain (that is, five separate models: Models M2). In the third stage, childhood exposures which were statistically significant at the 10% level in Model 2 were included together in a fully adjusted model which considered all domains together (Model 3). Finally, we adjusted for adult social class. For childhood exposures with 3 or more levels, a graded association with wellbeing was assumed and exposures were entered into regression models as continuous independent variables. All those with a valid WEMWBS score were included in regression models using Full Information Maximum Likelihood estimation to reduce the impact of missing covariate data. Adjustment was made for gender, as is customary, although there was no evidence of gender differences in WEMWBS scores and no evidence of interaction between gender and any of the childhood environment indicators, with one exception. We found a statistically significant interaction between gender and paternal care and this interaction term was included in all models where a main effect of paternal care was included. Stata 12.1 (SE) was used for all statistical analysis (StataCorp. 2011. *Stata Statistical Software*: *Release 12*. College Station, TX: StataCorp LP).

A total of 2661 (84.1%) of the target sample living in the UK and not having previously permanently withdrawn or lost contact participated in 2006–11 and 1976 of these had complete WEMWBS scores and were included in the analyses. The WEMWBS was not included at the start of data collection (n = 229) and the remaining 456 either did not take part in the assessment at which the instrument was administered or did not complete the WEMWBS instrument. Compared with those who provided wellbeing data at age 60–64, those who did not had more socioeconomically disadvantaged childhoods, were breastfed, had lower parental interest in their education, had less practical care of the house and child, had more overprotective fathers, had mother’s with poorer health, had lower birth weight, and were less frequently happy/sociable children. There were no differences in parental age at study member’s birth, other indicators of parent-child relationship quality, the frequency with which they had experienced parental divorce or separation from the mother, frequency of residential or school moves, mother’s neuroticism, father’s health, or number of childhood illnesses.

## Results

Table 1 summarises the material and psychosocial circumstances in childhood of those who remained in the study and provided WEMWBS scores at age 60–64. The majority of study members had parents with only primary level education and almost one in five lacked household amenities on two or more occasions. Very few were born to teen fathers or mothers and parental divorce was experienced by just over one in twenty study members. Around one in ten study members were separated from their mother for four or more weeks and a similar proportion were absent from school for a total of ten or more weeks (not necessarily consecutively) due to illness. Mean paternal care was 22.2 (standard deviation 7.6), mean paternal overprotection was 11.8 (6.5), mean maternal care 24.9 (6.6) and mean maternal overprotection was 12.8 (6.5).

**Table 1:** Childhood circumstances among members of the MRC National Survey of Health and Development who provided mental wellbeing data in 2006–11.

FAMILY SOCIOECONOMIC CIRCUMSTANCES		CHILDREARING AND PARENTING		PARENTAL HEALTH AND ADJUSTMENT	
Father’s social class	n (%)	Ever breastfed	n (%)	Father’s perceived health	n (%)
I	140 (7.1)	Yes	1460(78.3)	Excellent/good	1188 (69.8)
II	336 (17.0)	No	404 (21.7)	Average	329 (19.3)
IIINM	377 (19.1)		total n = 1864	Not good/father died	184 (10.8)
IIIM	540 (27.3)	**Practical care of house and child**			total n = 1701
IV	369 (18.7)	High	743 (43.7)	**Mother’s perceived health**	
V	96 (4.9)	Medium	475 (27.9)	Excellent/good	1172 (69.1)
	total n = 1858	Low	482 (28.4)	Average	428 (25.1)
**Father’s education**			total n = 1700	Not good/mother died	98 (5.8)
Secondary +degree/diploma	188 (10.8)	**Parental interest in child’s education**			total n = 1695
Secondary	303 (17.3)	High	497 (37.3)	**Maternal neuroticism**	**mean (sd)**
Below Secondary	325 (18.6)	Medium	460 (34.5)		1.56 (1.58)
Primary only	931 (53.3)	Low	375 (28.2)		
	total n = 1747		total n = 1332	**CHILDHOOD WELLBEING**	
**Mother’s education**		**Parental bonding instrument**	**mean (sd)**	**Low birthweight**	
Secondary +degree/diploma	128 (7.3)	Maternal care	24.9 (6.6)	No	1906 (96.8)
Secondary	288 (16.3)	Maternal overprotection	12.8 (6.5)	Yes	64 (3.3)
Below Secondary	331 (18.8)	Paternal care	22.2 (7.6)		total n = 1970
Primary only	1016 (57.6)	Paternal overprotection	11.8 (6.5)	**Teacher-rated happy/sociable child**	
	total n = 1763			High	214 (13.1)
**Occasions lack household amenities**		**FAMILY INSTABILITY**		Medium	1332 (81.8)
0	861 (53.5)	**Parental divorce**	**n (%)**	Low	83 (5.1)
1	456 (28.3)	No	1865 (94.4)		total n = 1629
2	272 (16.9)	Yes	111 (5.6)	**Childhood illnesses (weeks absent from school)**	
3	20 (1.20		total n = 1976	<2 weeks	364 (24.1)
	total n = 1609	**Separation from mother**		<4 weeks	437 (29.7)
**Teen father**		<4 weeks	1567 (88.7)	<6 weeks	510 (33.7)
No	1722 (99.3)	4+ weeks	199 (11.3)	<10 weeks	256 (17.0)
Yes	12 (0.7)		total n = 1766	10+ weeks	153 (10.1)
	total n = 1734	**Residential moves by age 16**			total n = 1511
**Teen mother**		0	563 (24.3)		
No	1696 (97.8)	1–3	1287 (69.2)		
Yes	39 (2.3)	4–8	121 (6.5)		
	total n = 1735		total n = 1861		
		**Junior school moves**			
		0	1145 (64.4)		
		1	231 (13.0)		
		2–4	401 (22.6)		
			total n = 1777		

**Notes:** mean (sd) = mean (standard deviation)

In bivariate models, 13 out of the 24 childhood indicators were associated with WEMWBS at the 10% level of statistical significance (Table 2, models M1). In a model considering all indicators of the family socioeconomic circumstances in childhood simultaneously, those born to teenage fathers were found to have substantially lower WEMWBS scores (-5.55 (95% CI -10.69, -0.41) than those born to fathers aged twenty or over, adjusting for all other childhood socioeconomic indicators (Table 2, models M2). No other childhood socioeconomic indicators were independently related to wellbeing. In the childrearing and parenting domain, those with more caring fathers had higher WEMWBS scores though there was evidence of a gender interaction with women benefitting less than men from higher paternal care (Table 2, models M2). WEMWBS scores were lower for those whose mothers were rated as providing poorer quality care of the house and child. There was a suggestion that greater levels of maternal overprotection were associated with lower WEMWBS scores (0.05<p<0.10). Indicators of family stability were not related to wellbeing (Table 2, models M2). Among the parental health and adjustment indicators, greater maternal neuroticism was negatively associated with WEMWBS scores and there was a trend towards lower WEMWBS scores for those with poorer maternal perceived health (Table 2, models M2). In the childhood wellbeing domain, children rated as more happy/sociable had higher WEMWBS scores (Table 2, models M2). Absence from school due to childhood illness was also associated with lower WEMWBS scores. Study members who were absent from school for ten weeks or more had poorer wellbeing (regression coefficient -1.15 (95% CI -2.03, -0.27) compared with those who had less than two weeks absence.

**Table 2:** Mutually adjusted associations between Warwick-Edinburgh Mental Well-Being Scores and domains of socioeconomic and psychosocial circumstances in childhood from multiple linear regression models.

		Models M1^a^	Models M2^b^	Model 3^c^
		sex-adjusted	mutually adjusted within domain	fully adjusted
	Reference group	Unstandardized regression estimate (95% CI)	Unstandardized regression estimate (95% CI)	Unstandardized regression estimate (95% CI)
**Family socioeconomic circumstances**				
Father’s social class age 4	per 1 class decrease	-0.18 (0.14)	-0.09 (0.18)	
Father’s education	per 1 level increase	0.22 (0.18)	0.21 (0.26)	
Mother’s education	per 1 level increase	0.03 (0.20)	-0.29 (0.25)	
Occasions lack amenities	per 1 unit increase	-0.18 (0.25)	-0.07 (0.27)	
Household crowding quintile	per 1 quintile increase	*-0*.*23 (0*.*14)*	-0.22 (0.16)	
Teen father	father at age 20+	*-4*.*00 (2*.*34)*	**-5.55 (2.62)**	-2.10 (2.29)
Teen mother	mother at age 20+	0.48 (1.31)	2.08 (1.47)	
**Childrearing and parenting**				
Never breastfed	ever breastfed	0.14 (0.45)	0.23 (0.44)	
Maternal care	per 1 unit increase	**0.16 (0.03)**	0.00 (0.04)	
Maternal overprotection	per 1 unit increase	**-0.18 (0.03)**	*-0*.*07 (0*.*04)*	**-0.12 (0.03)**
Paternal care	per 1 unit increase	**0.30 (0.04)**	**0.25 (0.05)**	**0.25 (0.04)**
Paternal care x female gender	per 1 unit increase	**-0.13 (0.05)**	**-0.14 (0.06)**	**-0.11 (0.05)**
Paternal overprotection	per 1 unit increase	**-0.22 (0.03)**	-0.08 (0.06)	
Parental interest in child’s education tertile	per 1 tertile increase	0.19 (0.28)	0.02 (0.29)	
Practical care of house and child	per 1 level decrease	**-0.72 (0.23)**	**-0.59 (0.25)**	*-0*.*41 (0*.*23)*
**Family instability**				
Parental divorce	no divorce	-0.14 (0.79)	-0.11 (0.80)	
Separation from mother before age 6	no separation	*-1*.*02 (0*.*61)*	-1.00 (0.61)	
Residential moves	per 1 additional move	-0.19 (0.15)	-0.17 (0.18)	
Junior school moves	per 1 additional move	-0.05 (0.15)	0.06 (0.17)	
**Parental health and adjustment**				
Father’s perceived health	per 1 level decrease	**-0.28 (0.13)**	-0.17 (0.16)	
Mother’s perceived health	per 1 level decrease	**-0.48 (0.16)**	*-0*.*33 (0*.*18)*	-0.23 (0.16)
Maternal neuroticism	per 1 unit increase	**-0.47 (0.13)**	**-0.40 (0.13)**	**-0.28 (0.13)**
**Childhood wellbeing**				
Low birthweight	birthweight 2500g+	-1.65 (1.03)	-1.47 (1.03)	
Happy/sociable child	per 1 level increase	**1.13 (0.47)**	**1.09 (0.47)**	0.74 (0.46)
Childhood illnesses	per 1 level increase	**-0.24 (0.09)**	**-0.23 (0.09)**	**-0.19 (0.09)**

**Notes:** Based on n = 1976 study members using Full Information Maximum Likelihood model; bolded text indicates p<0.05; italicised text indicates p<0.10.

^a^In models M1, each childhood exposure were included separately (sex-adjusted bivariate analysis).

^b^In models M2, all childhood exposures within a domain were included together.

^c^In model 3, all childhood exposures significant at p<0.10 in model 2 were included together.

When all five domains were considered together, four indicators of childhood circumstances were found to be associated with WEMWBS scores at the 5% level of statistical significance. Paternal care was positively associated with WEMWBS scores, and maternal overprotection, maternal neuroticism and absence from school due to childhood illness were negatively associated with WEMWBS scores (Table 2, model3). These associations were not attenuated on adjustment for adult social class, although WEMWBS scores were lower among those with more disadvantaged adult social class (Table 3). Table 3 additionally shows the standardised (beta) regression estimates that can be used to compare effect sizes across the different childhood indicators. For a one standard deviation increase in paternal care, there was an estimated 0.237 standard deviation increase in wellbeing. The effect sizes for maternal overprotection, maternal neuroticism, absence for childhood illness, and adult social class were smaller than this and of broadly the same magnitude as each other.

**Table 3:** Unstandardized and standardized fully adjusted model showing associations between Warwick-Edinburgh Mental Well-Being Scores and domains of socioeconomic and psychosocial circumstances in childhood.

	Unstandardized regression estimate (s.e.)	Standardized regression estimates
**Family socioeconomic circumstances**		
Teen father	-1.89 (2.28)	-0.019
**Childrearing and parenting**		
Maternal overprotection	**-0.12 (0.03)**	**-0.099**
Paternal care	**0.25 (0.04)**	**0.237**
Paternal care x female gender	**-0.12 (0.05)**	**-0.086**
Practical care of house and child	-0.27 (0.24)	-0.028
**Parental health and adjustment**		
Mother’s perceived health	-0.21 (0.16)	-0.033
Maternal neuroticism	**-0.28 (0.13)**	**-0.055**
**Childhood wellbeing**		
Happy/sociable child	0.70 (0.46)	0.036
Childhood illnesses	**-0.18 (0.09)**	**-0.050**
**Adult social class**	**-0.54 (0.14)**	**-0.085**

**Notes:** Based on n = 1976 study members using Full Information Maximum Likelihood model; bolded text indicates p<0.05

## Discussion

In this prospective study of men and women, childhood circumstances were associated with mental wellbeing fifty years later. Associations were not attenuated on adjustment for adult socioeconomic circumstances. In a model considering multiple domains of the childhood environment, elements of childrearing and parenting, parental health and adjustment and child wellbeing emerged as predictors of wellbeing at age 60–64 but family socioeconomic circumstances and family instability did not independently predict wellbeing.

Elements of the childhood environment that we identified as being relevant for later wellbeing were predominantly psychosocial in nature, with the exception of school absence due to illness. Elements of parent-child relationship quality, captured by the parental bonding instrument, were related to wellbeing. This is in line with previous analysis at earlier ages in this cohort with respect to women’s mental wellbeing [23] and psychological distress in men and women [29]. Maternal neuroticism, captured when the study member was age 15, was negatively correlated with wellbeing. It is possible that more neurotic mothers may signal to their offspring that everyday situations are threatening and difficult to cope with. There may also be a heritable component of maternal neuroticism, and indeed of parental personality characteristics, that is related to the other childhood exposures studied here. Personality factors, including neuroticism, are highly correlated with wellbeing [23,40]. Children who missed more schooling due to illness had poorer wellbeing and this was not explained by adult socioeconomic position. This association could indicate continuity of poor health from childhood through to adulthood since there is some evidence that wellbeing is lower among those with chronic conditions [41] although that evidence is somewhat mixed.

The lower wellbeing of children born to teen fathers was explained by other childhood exposures, notably childrearing and parenting. The lack of association between other elements of childhood socioeconomic circumstances and later wellbeing stands in contrast to previous work. The difference may be due to the different outcomes of interest since previous studies used life satisfaction [19,24] and self-esteem [18]. It is also interesting to note the lack of association between parental divorce and mental wellbeing since evidence from this cohort at earlier ages and in many other studies show considerably greater risk of psychological disorder amongst those who experienced parental divorce [7,28]. Whilst wellbeing is comprised of multiple dimensions that are positively correlated and to some extent overlapping, it appears that its different dimensions may be related to different determinants [42].

### Limitations

The WEMWBS instrument captures mental wellbeing over the previous two week period. For the current study, mental wellbeing assessed over a longer period may have been preferable although we note that test-retest reliability was high over a one week period [37]. We were not able to separate exposures in early childhood and adolescence although we have captured cumulative exposures where possible (for example crowding was measured on five occasions and household amenities on two). We measured a wide range of childhood exposures but some domains were less well measured. In particular, we had no data on parental substance use or father’s mental health. The quality of the parent-child relationship was captured retrospectively at age 43 but correlates in the expected direction with prospectively assessed childhood variables in this cohort [29] and the parental bonding instrument shows a high level of agreement between twins and other siblings in other studies [43,44], thus providing some validation for its retrospective administration. Study members who were not included in the current analysis tended to come from more socioeconomically disadvantaged families though excluded study members did not consistently differ on indicators on other domains of childhood experiences. In further analysis (available on request), we assessed whether the association between childhood experiences and wellbeing may be different among those who were lost to follow-up or did not provide WEMWBS data for other reasons. To do this, we examined the association between childhood happiness/sociability (which is plausibly an indicator of childhood mental wellbeing [45]) and each of the childhood exposures (one at a time) and included the interaction of each exposure and whether the study member provided WEMWBS data or not. This revealed an association between two indicators of family socioeconomic circumstances (namely childhood social class and household crowding) and childhood happiness/sociability among those who did not provide WEMWBS data but not among those who did provide WEMWBS data (p-value for interaction terms <0.05 in both models). This could suggest that attrition in the current study has resulted in some underestimation of the importance of family socioeconomic environment for mental wellbeing in later life.

### Conclusions

Elements of childrearing and parenting are associated with offspring’s wellbeing in early older age, with effects sizes that are larger or comparable to that of socioeconomic position in adulthood. Initiatives to improve the nation’s mental wellbeing (ONS) that include programmes targeted to supporting families and children may additionally have benefits that continue into older age.
